# IOTA simple rules: An efficient tool for evaluation of ovarian tumors by non-experienced but trained examiners - A prospective study

**DOI:** 10.1016/j.heliyon.2024.e24262

**Published:** 2024-01-07

**Authors:** Nguyet Dang Thi Minh, Thi Nguyen Van, Huu Duong Duc, Minh Nguyen Tuan, Giang Duong Thi Tra, Dat Do Tuan, Duc Nguyen Tai

**Affiliations:** a Department of Obstetrics and Gynecology, Hanoi Medical University, 1 Ton That Tung Street, Dong Da District, Hanoi, 100000, Viet Nam; b Department of Quan Su Radiology, K Hospital 43 Quan su Street, Hoan Kiem district, Hanoi, 100000, Viet Nam; c Department of Delivery, Hanoi Obstetrics and Gynecology Hospital, 929 La Thanh Street, Ba Dinh district, Hanoi, 100000, Viet Nam; d Prenatal screening and diagnostic center, Hanoi Obstetrics and Gynecology Hospital, 929 La Thanh Street, Ba Dinh district, Hanoi, 100000, Viet Nam

**Keywords:** IOTA simple rules, Ovarian tumor, Non-expert examiner, Training, Vietnam

## Abstract

**Objectives:**

A simple and efficient tool for evaluating ovarian tumors in general hospitals where radiologists without experience in gynecological ultrasound is necessary. This study aims to evaluate the diagnostic performance of IOTA simple rules in initial classification of ovarian tumors by non-experienced examiners who have received simple training.

**Materials and method:**

A prospective single-center study was conducted at Hanoi Obstetrics and Gynecology Hospital. Three resident gynecologists trained themselves for two weeks and then received hands-on practice under the supervision of experts for another two weeks. The examiners performed ultrasound on 424 eligible women scheduled for surgery for ovarian tumors and classified the tumors based on IOTA simple rules. The postoperative pathology of ovarian tumors was used as the gold standard.

**Results:**

90.8 % (385/424) of the tumors were benign. Simple rules were applicable in 399/424 (94.1 %) tumors, with a sensitivity of 84.8 % (95 % CI, 70.2–94.3), specificity of 98.9 % (95 % CI, 97.5–99.7), positive predictive value of 87.5 % (95 % CI, 73.3–95.9), and negative predictive value of 98.6 % (95 % CI, 97.1–99.5). The sensitivity of IOTA simple rules was higher in postmenopausal women (91.7 % vs. 81.0 %), while the specificity was higher in premenopausal women (99.4 % vs. 95.8 %). Accuracy was 100 % in all ten pregnant women were assessed using these rules.

**Conclusion:**

In conclusion, in the hands of non-expert examiners who were trained thoroughly, IOTA simple rules are a simple and efficient tool for clinical practice in centers where expert radiologists in gynecology are not always available. The training program is simple and could be applied widely in other clinical centers. Further studies are necessary to evaluate the effectiveness of the IOTA simple rules in assessing ovarian tumors among pregnant women.

## 1 Introduction

Ovarian tumors are common gynecological diseases and have a specific malignancy rate. According to Torre et al., Asian/Pacific Islander women are one of four groups with the highest ovarian cancer prevalence (9.2 per 100,000) [1]. The initial evaluation of ovarian tumors plays an essential role in categorizing patients and planning treatment. Among the many modern diagnostic imaging techniques, ultrasound continues to be a helpful tool and the first choice for identifying and characterizing adnexal masses [2].

To date, many preoperative classification systems using sonography to characterize ovarian tumors have been published, such as International Ovarian Tumor Analysis (IOTA) simple rules (2008) [3], Gynecologic Imaging-Reporting and Data System (GI-RADS, 2009) [4], and Ovarian-Adnexal Imaging-Reporting-Data System (ORADS, 2018) [5]. In 2021, a Consensus Statement on the preoperative diagnosis of ovarian tumors by ESGO/ISUOG/IOTA/ESGE was published, requiring the participation of expert gynecological radiologists or the use of the IOTA ADNEX model, which may be relatively complicated for non-experienced examiners in gynecological ultrasound [6]. Meanwhile, most patients with ovarian tumors are asymptomatic and usually detected incidentally during imaging for another indication [7]. The diagnosis of ovarian tumors is generally made in general hospitals rather than in Obstetrics and Gynecology or Oncology hospitals [8]. Therefore, there is a need for a simple and effective tool for non-expert examiners in the initial evaluation of ovarian tumors.

IOTA simple rules were proposed by the International Ovarian Tumor Analysis (IOTA) group in 2008. IOTA simple rules use five ultrasound features indicative of a benign tumor (B-features) and five ultrasound features indicative of a malignant tumor (M-features). Based on which of the B- and M-features apply, tumors are classified as Benign (Only B-features apply), Malignant (Only M-features apply), or Inconclusive (no features apply, or both B- and M-features apply). The efficiency of IOTA simple rules in evaluating ovarian tumors has been demonstrated in previous studies with sensitivity and specificity of approximately 93 % and 95 %, respectively [8]. However, almost all studies were conducted by experts or experienced examiners in gynecological ultrasound [[9], [10], [11]]. To date, some studies have been performed to evaluate ovarian tumors according to IOTA simple rules by non-expert examiners, but they did not mention whether there was training or not [12,13]. In 2015, Tinnangwattana et al. conducted a study to evaluate the diagnostic performance of IOTA simple rules in predicting malignant adnexal tumors by non-expert examiners. The residents were trained for IOTA simple rules by the experienced examiner (TT), using 50 teaching video clips for two weeks and then hands-on practice under supervision for 20 cases. However, the source of training video clips was not cited clearly [14].

To find a simple and efficient tool for clinical practice in general hospitals where examiners do not have experience in gynecological ultrasound, we conducted this study to evaluate the value of IOTA simple rules in the initial classification of ovarian tumors by non-experienced examiners who were simple trained.

## 2 Materials and Method

This is a prospective single-center study conducted from August 2020 to August 2022 at Hanoi Obstetrics and Gynecology Hospital. The study was approved by the Ethics Committee of Hanoi Obstetrics and Gynecology Hospital (Number: HĐĐĐ/PSHN-1720)

### 2.1 Participants/eligibility criteria

We enrolled women admitted to the Hanoi Obstetrics and Gynecology Hospital for scheduled surgery due to ovarian tumors from December 2020 to May 2022. Exclusion criteria included women with emergency surgery due to torsion or ruptured ovarian tumors, recurrent ovarian cancer, ovarian tumors associated with other cancers, and pregnant women less than 12 weeks pregnant. We did not exclude pregnant women over 12 weeks of gestation for two reasons. First, pregnant women still require surgery for ovarian tumors due to the risk of torsion, labor obstruction, or malignancy. Second, ultrasound does not pose any risk to the mother or fetus.

### 2.2 Ultrasound examination

Phase I: Three resident gynecologists with less than one year of experience in gynecological ultrasound were trained for IOTA simple rules by themselves, using articles [3,15] and educational materials & lectures from the International Ovarian Tumor Analysis Group's website (https://iota.education/) for two weeks. They then received hands-on practice under an expert's supervision for another two weeks.

Phase II: Eligible women were informed about the study by gynecologists and signed an agreement form to participate. Participants' baseline characteristics, including age, BMI, and menstruation status, were collected. For women who had undergone a hysterectomy (due to fibroid or placenta percreta), postmenopausal status was defined as having an age of ≥50 years. Afterward, they were transferred to the ultrasound room and evaluated for ovarian tumors by one of three examiners. The examiners were blind to the patient's history and previous ultrasound results. Participants underwent ultrasound examination using either transabdominal, transvaginal, or both approaches, as appropriate. The GE Voluson S6 Ultrasound Machine with real-time 2.5–5 MHz or 5–7.5 MHz transvaginal curvilinear transducer was used. The morphology and vascularization of ovarian tumors were evaluated and described based on IOTA simple rules. The ovarian tumors were categorized into one of three groups: Benign (only B-features apply), Malignant (only M-features apply), or Inconclusive (no features apply, or both B- and M-features apply). The larger tumor was chosen for evaluation if patients had bilateral ovarian tumors. The size of tumors was defined according to the largest diameter measured during the ultrasound examination.

**Fig. 1:**
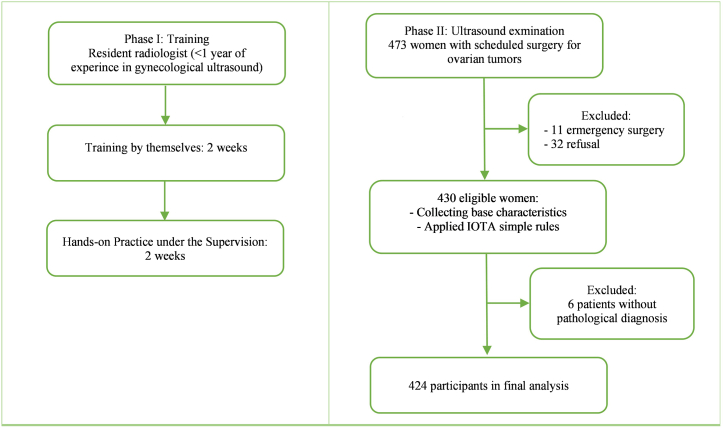
Patient accrual flowchart.

The postoperative pathology of ovarian tumors was used as the gold standard. Pathologists were only aware of the results of the patients' preoperative regular ultrasound and were blinded to the category of IOTA. Patients without a histological diagnosis were excluded from the final analysis. The criteria recommended by the International Federation of Gynecology and Obstetrics were used to classify ovarian tumors according to the local protocol [16]**.** As in many previous studies, borderline ovarian tumors were considered malignant due to the risk of invasion. Fig. 1 presents a flowchart.

### 2.3 Statistical analysis

SPSS version 26 was used for statistical analysis.

The sensitivity, specificity, positive predictive value, negative predictive value, positive likelihood ratio, and negative likelihood ratio of simple rules and pattern recognition were calculated. These analyses were based on the 399 patients to whom the simple rules were applicable.

## 3 Results

During the research period, four hundred and seventy-three women were admitted for surgery for ovarian tumors. Excluding forty-nine patients (eleven who underwent emergency surgery, thirty-two who refused to participate, and six who had no postoperative pathology results), we had four hundred and twenty-four participants for the final analysis. The average age of the participants was 38 years (range 12–81). Ten women (2.4 %) were pregnant, and sixty-six were postmenopausal (15.6 %). Approximately three-fourths of the tumors were less than 10 cm.

Among the 424 women in the final analysis, 362 underwent laparoscopic surgery, 57 underwent laparotomy, and five conversion from laparoscopy to laparotomy due to abdominal adhesions. There were no operation-related complications.

In terms of pathology, more than 90 % (385/424) of tumors were benign, and the proportion of borderline and malignant tumors was 1.9 % and 7.3 %, respectively. Overall, IOTA simple rules were applicable in 399/424 (94.1 %) tumors, with a sensitivity and specificity of 84.8 % (95 % CI, 70.2–94.3) and 98.9 % (95 % CI, 97.5–99.7), respectively. The probability that an ovarian tumor with a positive result according to IOTA simple rules was malignant is 87.5 % (95 % CI, 73.3–95.9), and that an ovarian tumor with a negative result was benign is 98.6 % (95 % CI, 97.1–99.5) (Table 1, Table 2.).

**Table 1:** Demographics of study population.

Demographics	n	Value
Age (mean ± SD)	424	38.5 ± 13.12
Number of living births (%)		
0	114	26.9
1	76	17.9
2	174	41
3	51	12
4	8	2.2
Marital status (%)		
Married	346	81.6
Single	78	18.4
Menstruation (%)		
Pre-menopause	358	84.4
Post-menopause	66	15.6
Pregnancy (%)		
Pregnant	10	2.4
Non-pregnant	414	97.6
Size (mean ± SD; mm)	424	87.6 ± 45.4
Location (%)		
Left	214	50.5
Right	210	49.5
Surgical methods (%)		
Laparoscopy	362	84.5
Laparotomy	57	13.4
Conversion from laparoscopy to laparotomy	5	1.2

As expected, the prevalence of malignant tumors was much higher in postmenopausal women (21.2 %) than in premenopausal women (4.7 %). IOTA simple rules were applied in 94.7 % (320/358) of premenopausal women and 90.1 % of postmenopausal women (47/66). The sensitivity of IOTA simple rules is higher in postmenopausal women (91.7 % vs. 81.0 %); in contrast, specificity is higher in premenopausal women (99.4 % vs. 95.8 %). Regarding pregnancy, IOTA simple rules could use in all 10 cases with accuracy being 100 %. One of them had a mucinous borderline tumor (Table 2, Table 3.).

**Table 2:** Rule applications of IOTA simple rules in evaluation ovarian tumors.

Study population	Rules applicable n (%)	IOTA simple rule			Pathology		
		Benign n (%)	Malignant n (%)	Inconsecutive n (%)	Benign n (%)	Malignant n (%)	Borderline n (%)
Total (n = 424)	399 (94.1)	367 (86.6)	32 (7.5)	25 (5.9)	385 (90.8)	31 (7.3)	8 (1.9)
Menstruation							
Pre-menopause (n = 358)	340 (94.7)	320 (89.4)	19 (5.3)	19 (5.3)	334 (93.3)	17 (4.7)	7 (2)
Post-menopause (n = 66)	59 (90.1)	47 (71.2)	13 (19.7)	6 (9.1)	51 (73.3)	14 (21.2)	1 (1.5)
Pregnancy							
Pregnant (n = 10)	10 (100)	9 (90)	1 (10)	0 (0)	9 (90)	0 (0)	1 (10)
Non-pregnant (n = 414)	389 (93.9)	358 (86.5)	31 (7.5)	25 (6.0)	376 (90.8)	31 (7.5)	7 (1.7)

**Table 3:** Accuracy of IOTA simple rules (n = 399).

IOTA simple rules	Pathology		Sensitivity % (95%CI)	Specificity % (95%CI)	PPV % (95%CI)	NPV % (95%CI)	LR+ % (95%CI)	LR- % (95%CI)
	Malignant	Benign						
Total								
Positive	28	4	84.8 (70.2–94.3)	98.9 (97.5–99.7)	87.5 (73.3–95.9)	98.6 (97.1–99.5)	77.6 (28.9–207.9)	0.15 (0.07–0.34)
Negative	5	362						
Menstruation								
Pre-menopause								
Positive	17	2	81.0 (61.1–93.7)	99.4 (98.1–99.9)	89.5 (70.9–98.2)	98.8 (97.1–99.6)	128 (31–520)	0.19 (0.08–0.46)
Negative	4	316						
Post-menopause								
Positive	11	2	91.7 (68.1–99.5)	95.8 (87.7–99.3)	84.6 (59.6–97.3)	97.9 (91.0–99.9)	22 (5.6–86.3)	0.09 (0.01–0.57)
Negative	1	46						

Among the five false-negative tumors when IOTA simple rules were applied, four cases were borderline tumors, and one was a Stage I ovarian cancer.

Of the tumors where IOTA simple rules could not be applied (25/424), 76 % (19/25) were benign. Approximately half of these inconclusive classifications were mucinous tumors (12/25), with malignant prevalence being 25 % (3/12), and all seven endometrioid tumors (28 %) were benign (Table 4).

**Table 4:** Pathology results for inconclusive according to IOTA simple rules.

Type of tumors	n	Pathology		
		Benign	Malignant	Borderline
Serous tumor	**6**	3	1	2
Mucinous tumor	**12**	9	1	2
Endometrioid tumors	**7**	7	0	0
Total	**25**	19	2	4

## 4 Discussion

Initial diagnosis has a significant effect on the management strategy of ovarian tumors, particularly among women at the age of childbearing. According to an updated consensus, which was the result of a Delphi study, surgery and chemotherapy are used for all patients with ovarian cancer [17]. Obviously, these treatments seriously affect the fertility of women. Among women who desire pregnancy in the future, oocyte cryopreservation should be discussed before starting treatment in a tertiary hospital where coordination involves gynecological oncology and assisted reproduction. In our study, we aimed to evaluate the value of IOTA simple rules in the initial classification of ovarian tumors by non-experienced examiners who received thorough training at a minimum cost. The performance of our young examiners was remarkable. We found that IOTA simple rules could be applied in 94.1 % (399/424) of tumors, with sensitivity and specificity being 84.8 % and 98.9 %, respectively.

Previous studies have evaluated the value of IOTA simple rules in the assessment of adnexal tumors by non-expert examiners. Alcázar et al. (2013) showed that the IOTA simple rules could be applied in approximately 80 % (270/324), with sensitivity, specificity, LR+, and LR-being 87.9 % (95 % CI, 72.4–95.2), 97.5 % (95 % CI, 94.6–98.8), 34.7 (95 % CI, 15.6–77.3), and 0.12 (95 % CI, 0.05–0.31), respectively [12]. The three-step strategy was efficient in discriminating between benign and malignant adnexal masses [13]. Of the 218 tumors that could not be classified using simple descriptors, 147 (67.4 %) were classified using simple rules. Within the second step, the respective values for sensitivity, specificity, LR+, and LR-were 44.8 % (95 % CI, 27.7–62.8), 99.2 % (95 % CI, 96.3–100), 52.9 (95 % CI, 7.2–388.1), and 0.6 (95 % CI, 0.4–7.7). The number of malignant tumors could be blamed. Our higher results might be explained by the lower proportion of malignant tumors that are usually more complicated and challenging to categorize than benign tumors. In our study, 9.2 % of tumors were malignant (2.4 % were borderline), while the malignancy rate in the above studies was higher, at 16.2 % (55/340) and 19.7 % (29/147), respectively. Another notable point is that these studies did not mention training programs. An earlier study has emphasized the importance of training examiners in terminology and simple rules before introducing them into guidelines and daily clinical practice [18]. In 2013, Tinnangwattana et al. conducted a similar study in which Obstetrics/Gynecology residents were trained directly by experts for two weeks and received hands-on practice under supervision for 20 cases. The results showed comparable outcomes to ours, with 94 % of tumors being classified, and sensitivity and specificity values of 89.3 % and 83.3 %, respectively [14]. However, our training program focused on self-learning, leading to a lower cost.

In a meta-analysis including six original studies [8], the pooled sensitivity and pooled specificity of simple rules on the hands of experts were 93 % (95 % CI, 90–96 %) and 95 % (95 % CI, 93–97 %), respectively. Compared to the meta-analysis study, our false-negative rate was relatively higher (15.2 % vs. 7 %). In our study, four out of five cases missed by simple rules were borderline. With rare and small internal papillary projections, thin septations without definite enhancement, and small size, these imaging features of borderline tumors close to benign rather than malignant characteristics [19]. This results in missed classification on ultrasound.

Although the exact numbers vary among previous studies, they reported a similar conclusion regarding IOTA simple rules in postmenopausal women. The sensitivity of the rules is higher, and the specificity is lower compared to premenopausal women [9,12,20]. Comparing to the meta-analysis study of Nunes et al., our study found that the rules were applied less frequently in premenopausal women (90.1 % vs. 94.7 %) [8]. The sensitivity of simple rules was higher in postmenopausal participants (91.7 % vs. 81 %), but the specificity was lower (95.8 % vs. 99.4 %). We also observed a higher prevalence of malignancy in postmenopausal women (20.3 %) than in premenopausal women (6.2 %). These results support the hypothesis that when simple rules are applicable, sensitivity increases, but specificity decreases with the increasing proportion of malignant tumors in the study population [8].

To date, there is no consensus about the diagnosis and management of adnexal masses among pregnant women. The key challenges in assessing ovarian tumors during pregnancy include: 1) using Color Doppler in the first trimester could pose a potential theoretical risk to the embryo, and 2) the features in simple rules related to Color Doppler may not be accurately applied due to the rapid changes in Doppler indices during pregnancy [21]. Although the number of pregnant women in our study was limited, the results have shown optimistic outcomes with all ten masses being correctly classified by simple rules. Therefore, further studies on the diagnostic performance of IOTA simple rules in pregnant women are necessary.

The strength of this study is that young doctors received thorough training, focusing on self-learning ability. This training program is simple and easy to use in other clinical practice centers. Furthermore, examiners directly performed ultrasound before surgeries, which blinded them to pathology results, thus limiting bias factors.

We acknowledge some limitations of our study. Firstly, due to a staffing shortage, we were unable to perform a three-step approach with expert participation throughout the entire study period. However, it is a fact that in clinical practice, radiologists typically work independently, and experts are not present in all cases. Secondly, we only included tumors that required surgery. Nevertheless, this issue was unlikely to affect the results because tumors that did not require surgery were usually simple and could be easily classified.

## 5 Conclusion

In conclusion, in the hands of non-expert examiners who were trained thoroughly, IOTA simple rules are a simple and efficient tool for clinical practice in centers where expert radiologists in gynecology are not always available. The training program is simple and could be applied widely in other clinical centers. Further studies are necessary to evaluate the effectiveness of the IOTA simple rules in assessing ovarian tumors among pregnant women.

## Declarations

The study was approved by the Ethics Committee of Hanoi Obstetrics and Gynecology Hospital (Number: HĐĐĐ/PSHN-1720)

This research did not receive any specific grant from funding agencies in the public, commercial, or not-for-profit sectors.

There are no conflicts of Interest concerning any of the authors.

## Data availability statement

Duong, Giang (2023), “IOTA-Simple rules - Vietnam”, Mendeley Data, V1,

doi: 10.17632/bsn8sdh48r.1

## CRediT authorship contribution statement

**Nguyet Dang Thi Minh:** Writing – review & editing, Supervision, Methodology, Formal analysis, Conceptualization. **Thi Nguyen Van:** Writing – review & editing, Writing – original draft, Methodology, Formal analysis, Conceptualization. **Huu Duong Duc:** Writing – original draft, Methodology, Formal analysis, Data curation, Conceptualization. **Minh Nguyen Tuan:** Methodology, Formal analysis, Data curation, Conceptualization. **Giang Duong Thi Tra:** Writing – review & editing, Writing – original draft, Methodology, Formal analysis, Data curation, Conceptualization. **Dat Do Tuan:** Methodology, Formal analysis, Data curation, Conceptualization. **Duc Nguyen Tai:** Methodology, Formal analysis, Data curation, Conceptualization.

## Declaration of competing Interest

The authors declare that they have no known competing financial interests or personal relationships that could have appeared to influence the work reported in this paper.
